# The Effect of Semaglutide With Lifestyle Intervention on the Physical Health of Patients Treated With Antipsychotic Drugs in a Secure Mental Health Setting: Protocol for an Uncontrolled Pretest-Posttest Pilot Mixed Methods Study

**DOI:** 10.2196/76013

**Published:** 2025-12-09

**Authors:** Kristina Brenisin, Florence Kinnafick, James King, Louise Millard, Donna Arya, Elizabeth Hodgson, Michelle Huggins, Andrew Simmons-Roberts, Martin O'Dowd, Nick Rayment, Parul Shah, Kieran C Breen

**Affiliations:** 1 St Andrew's Healthcare, Billing Road, Northampton, NN1 5DG, United Kingdom, +44 1604 616049; 2 National Centre for Sport and Exercise Medicine, School of Sport, Exercise and Health Sciences, Loughborough University, Loughborough, United Kingdom

**Keywords:** antipsychotics, semaglutide, obesity, glucagonlike peptide-1, GLP-1, body weight loss, diabetes, severe mental illness

## Abstract

**Background:**

Antipsychotic-induced weight gain is a common side effect of antipsychotic drug treatment, particularly with second-generation medications such as clozapine and olanzapine. Weight gain in patients undergoing antipsychotic therapies is a significant concern, often compounded by factors related to their condition that can be particularly challenging in a secure care setting. While there is significant evidence to support the benefits of semaglutide, one of the available glucagon-like peptide-1 receptor agonists, in promoting weight loss for those who have a general weight-related health issue and meet the referral criteria for specialist services, it is unclear whether it will be as successful in people who have specifically gained weight due to medication-associated side effects and who reside in a secure care setting.

**Objective:**

This study aims to assess the impact of semaglutide in combination with a lifestyle behavior change intervention on the physical health measures of patients in a secure setting who have atypical antipsychotic–induced weight gain and identify enhancements to the intervention, specifically geared toward improving adherence and acceptability from both staff and patient perspectives.

**Methods:**

This 2-year uncontrolled pretest-posttest pilot study aims to recruit 20 inpatient participants. Adult patients of any diabetic status with a minimum BMI of 35.0 kg/m^2^ (or a BMI of 32.5 kg/m^2^ for people from South Asian, Chinese, other Asian, Middle Eastern, Black African, or African-Caribbean descent) who are receiving inpatient treatment and are treated with either olanzapine or clozapine will be eligible for inclusion in the study. Patients will receive semaglutide (Wegovy) at a maintenance dose of 2.4 mg once a week for 2 years. All participants will also receive a lifestyle behavior change intervention.

**Results:**

The findings will reveal whether the format of the interventional approach is both sustainable and effective for adult patients diagnosed with severe mental illness and living with obesity who are currently residing in a secure mental health setting. Implementation changes that could improve the acceptability of and adherence to the intervention will be explored.

**Conclusions:**

This research should be beneficial for patients with severe mental illness who are living with obesity and are residing in a secure setting as the findings may ultimately reduce the mortality risk in this patient group.

## Introduction

### Background

Obesity represents a common clinical challenge among patients with severe mental illness (SMI) as it contributes to increased comorbidities [1] and reduces life expectancy by up to 25 years [2]. Many medications prescribed for SMIs, such as antipsychotics, are associated with weight gain, increased appetite, and metabolic changes that can complicate weight management [3,4]. Such side effects are especially prevalent with second-generation antipsychotics [5], also known as serotonin-dopamine antagonists, which are atypical for their lower risk of extrapyramidal side effects [6]. Notably, olanzapine and clozapine, both atypical antipsychotics, carry a particularly high risk of weight gain [7-12].

Behavioral lifestyle interventions incorporating modifications in diet and physical activity (PA) remain the foundation of treatment for overweight and obesity; however, they are often not associated with clinically meaningful and sustainable weight loss either in the general population [13] or in individuals with SMI [14]. There is evidence suggesting that, while people with SMI can engage in and benefit from lifestyle interventions, in general, greater support using a more tailored approach is needed [15,16]. In addition, lifestyle interventions still need to be translated into routine care [17] and improved for the management of antipsychotic-induced weight gain (AIWG) [18]. Furthermore, such interventions are not effective to prevent or treat adverse metabolic effects caused by antipsychotics [19]. Therefore, pharmacotherapy is recommended as an additional tool for long-term weight management in people with a BMI of at least 30 kg/m^2^ or at least 27 kg/m^2^ in those with weight-related comorbidities [20-22].

There is increasing evidence supporting pharmacological treatment for weight reduction with antiobesity drugs. While several antiobesity drugs are available [23], treatment with glucagon-like peptide-1 receptor agonists (GLP-1 RAs) for obesity management appears to be effective for and well tolerated by people with schizophrenia [24]. GLP-1 is an incretin hormone responsible for glucose regulation and satiety. It improves β-cell functioning and glucose tolerance and plays a crucial role in stimulating glucose-dependent insulin secretion [25,26]. As a result, GLP-1 RAs are being used increasingly to manage type 2 diabetes (T2D) by improving glycemic control [26,27]. Semaglutide is one of the GLP-1 RAs approved for treatment of T2D [28], and it has also been assessed for efficacy and safety in weight management among adults with overweight or obesity regardless of diabetic status [29-31] and evaluated in combination with behavioral interventions [32]. Treatment with semaglutide demonstrated superior, clinically meaningful, and sustained weight loss in patients with obesity (BMI of ≥30 kg/m^2^) or overweight (BMI of ≥27 kg/m^2^ to <30 kg/m^2^) who had at least one weight-related comorbidity without T2D in various Semaglutide Treatment Effect in People With Obesity (STEP) trials [33]. In addition to reduced weight, treatment with semaglutide also significantly improved waist circumference, cardiovascular functioning, and physical functioning compared to placebo [34].

While semaglutide shows benefits for weight-related health issues in the general population living with obesity [35,36], its effectiveness for AIWG in a specific psychiatric setting remains unclear. Siskind et al [24] demonstrated the effectiveness of GLP-1 RAs for weight loss in individuals treated with clozapine and olanzapine, but out of 168 participants, only 17.9% reported a decrease in BMI. However, only 3 studies were included in their review, where the treatment with GLP-1 RAs ranged from 12 to 24 weeks. A more recent systematic review conducted by Bak et al [37] also explored the effectiveness of GLP-1 RAs in AIWG, but only limited data were available. Semaglutide was not used in any of the studies, and furthermore, lifestyle interventions were not included in the trials despite evidence in the general semaglutide trials suggesting that a multifactorial approach improves clinical outcomes [32].

Prasad et al [38] explored semaglutide for the treatment of AIWG, and initial results suggested the potential effectiveness of the drug; however, a more rigorous trial is required. Specifically, only 2 out of 12 patients involved in their study were receiving clozapine and olanzapine, and the dose of semaglutide did not reach the dose approved for the management of obesity. Furthermore, 2 short-term (26-36 weeks) clinical trials have been registered to assess the effects of semaglutide on people prescribed clozapine or olanzapine [39,40]. However, these do not include dietary or PA components, not all participants will be residing in a psychiatric setting, and individuals with T2D or treated with corticosteroids or other hormone therapy are excluded from the study. Both studies exclude individuals that either are treated with diabetes medication or have been diagnosed with diabetes despite GLP-1 RAs demonstrating their efficacy for reducing obesity-related adverse health effects in adults regardless of obesity status [41].

### Study Objectives

The primary objective of this study is to evaluate the effectiveness of semaglutide (Wegovy) in combination with a lifestyle intervention. Specifically, this study explores whether body weight is reduced and, secondarily, whether other physical health outcomes, including change in waist circumference, BMI, blood pressure, resting heart rate, and blood cholesterol, are improved in patients with SMI who are treated with clozapine or olanzapine and are residing in a psychiatric setting. In addition, secondary outcomes will be assessed to understand the mechanism through which the intervention influences the patients’ overall lifestyle and contributes to changes in specific aspects of their physical and mental health. Therefore, an evaluation will be conducted to understand the effectiveness of the intervention, which has been modified from a previous STEP protocol for implementation in a secure mental health setting (STEP@STAH). The feasibility of the intervention, participant engagement, and factors that could influence the outcomes of the study will be explored. This study also aims to inform a future lifestyle intervention by exploring potential barriers and facilitators from both patient and staff perspectives.

## Methods

### Design

A 2-year uncontrolled pretest-posttest intervention study will be conducted in a secure mental health hospital that provides specialist mental health care for patients with some of the most complex and challenging mental health needs in the United Kingdom from both forensic and nonforensic pathways. The efficacy of semaglutide in combination with a lifestyle intervention for AIWG in individuals with SMI residing in a psychiatric setting who have been treated with clozapine or olanzapine will be evaluated.

This study has been designed for patients to receive semaglutide once a week. Participants will be offered a lifestyle behavior change intervention including dietary advice, PA advice, and psychological support according to their personalized care plan from the time of enrollment into the trial. The behavioral element of the intervention, which was designed by the hospital’s dietetics team, will be introduced by KB and supported on the ward by members of the care team. The program covers 24 nutrition and exercise topics and includes the provision of fact sheets, a food diary, and a workbook. Participants will also have access to an extensive range of support resources.

A mixed methods design will be used in the study to conduct an effective evaluation of the intervention. Quantitative data will be collected on patient outcomes during the 2-year intervention, and participants’ engagement with the 3 elements that will support the medication—psychological therapy, diet, and PA—will be explored. Qualitative data will be gathered to evaluate the intervention, specifically exploring its fidelity, effectiveness, acceptability, and implementation within a secure mental health setting. These data will be collected from both staff and patients to maximize feedback on the intervention.

### Participants

Up to 20 inpatient participants with SMI—with a minimum requirement of 5 participants—who are currently treated with either clozapine or olanzapine will be recruited for the study. A minimum of 5 is necessary to ensure that preliminary data are obtained and the feasibility of the intervention is assessed [42]. A maximum of 20 patients ensures that the study remains manageable in terms of monitoring and data collection and analysis while still providing sufficient diversity so that meaningful patterns are observed. Using a mixed methods approach, and considering the data that will be collected, the minimum sample size will provide valuable insights, whereas the maximum sample size will ensure the practicality of the pilot study. Inclusion and exclusion criteria for patients are listed in Textbox 1.

Textbox 1. **Eligibility criteria.**

**Inclusion criteria**

- Age ≥18 years
- BMI ≥35.0 kg/m^2^ (or ≥32.5 kg/m^2^ for people from South Asian, Chinese, other Asian, Middle Eastern, Black African, or African-Caribbean descent)
- Ability to provide informed consent as assessed by their responsible clinician
- Current prescription for olanzapine or clozapine
- Current residence in an inpatient setting
- Sufficient level of English to read and understand the information sheet (may require verbal explanation or support from staff)

**Exclusion criteria**

- Age ≤17 years
- Meeting any of the contraindications for semaglutide, including hypersensitivity to the active substance or to any of the excipients
- Breastfeeding
- Pregnancy or likelihood of becoming pregnant
- Diagnosis of dementia or Huntington disease
- Admission to a psychiatric intensive care unit or acute ward
- Discharge anticipated within 6 months’ time or less

The recruitment is clinician mediated, with random selection for prioritization in the event of a Wegovy shortage in the United Kingdom. Although a shortage is not anticipated, this step is included for completeness. In case prioritization is required, the random selection process will be conducted by the project manager, who will provide the selected participants’ ID to the pharmacy. All other participants will remain on the waiting list until the medication becomes available, but they will be expected to start taking part in the lifestyle intervention.

Responsible clinicians (RCs) conduct an informed consent process with the potential participants before formal screening. If the number of eligible participants exceeds the available medication supply, a final priority list will be randomly generated. RCs may delegate the informed consent process to the researcher or other suitable delegate, such as a specialty physician.

Potential participants are provided with a participant information sheet and are given at least 24 hours to consider whether they would like to take part.

After a minimum of 24 hours, the potential participant is reapproached by the RC to determine whether they wish to continue with the study. At this point, patients are given the opportunity to ask any questions they may have. If the patient agrees to be involved in the project, the RC gives them the consent form to sign.

Once the patient has provided informed consent, the RCs oversee the referral and screening process. This may be done in liaison with the pharmacy team to identify and assess any potential contraindications or caution for use, including diabetic retinopathy and history of pancreatitis, ensuring that only eligible participants can be enrolled in line with safety guidelines and prescribing recommendations.

Semaglutide is not considered a critical care medication for those enrolled in the trial; therefore, the random selection method, which does not prioritize any particular group of patients, is appropriate to maintain the trial’s scientific integrity. This approach ensures unbiased allocation, facilitating the cohort study design that will be used to evaluate the medication’s effectiveness across different patient groups and enhancing the validity of the study’s findings.

Patients on the medication waiting list will continue with the healthy lifestyle element of the intervention. This will allow for the assessment of the impact of the nonpharmaceutical element of the intervention on the patients’ BMI and overall physical health outcomes.

### Pharmacological Treatment

Semaglutide (Wegovy) injection is the medication that will be used in the trial. An induction dose of 0.25 mg, titrated up every 4 weeks to 0.5 mg, 1.0 mg, 1.7 mg, and 2.4 mg, will be administered, with a maintenance dose of 2.4 mg or up to the maximum tolerated dose. To reduce the likelihood of gastrointestinal symptoms, the dose should be escalated over a 16-week period to a maintenance dose of 2.4 mg once weekly. In case of significant gastrointestinal symptoms, delaying dose escalation or lowering to the previous dose until symptoms have improved will be considered. Weekly doses higher than 2.4 mg will not be administered as they are not approved for use.

If patients are unable to lose at least 5% of their initial body weight after 6 months of treatment, a clinical decision will be required on whether to continue treatment considering the benefit and risk profile of the individual patient. This decision will be made in consultation between the RC and the patient, with input from other members of the clinical team. It will also consider the patient’s adherence to the other elements of the intervention, including diet and PA. This 6-month evaluation is in accordance with the UK National Institute for Health and Care Excellence guidelines.

The semaglutide injection will be administered once weekly on the same day of the week throughout the study period. Injections will be administered in the thigh, abdomen, or upper arm at any time of the day irrespective of meals, with the injection site selected according to the patient’s preference. The medication will be administered by a member of the clinical care staff or by the participant with support if preferred and safe to do so. When administering the medication, the National Institute for Health and Care Excellence guideline TA875 [28] and standard hospital prescribing processes will be followed.

If a dose is missed, it should be administered as soon as possible and within 5 days after the missed dose. If more than 5 days pass, the missed dose should be skipped, and the next dose should be administered on the regularly scheduled day. In either case, patients can then resume their regular once-weekly dosing schedule. If more doses are missed, reducing the starting dose for reinitiation should be considered.

### Behavioral Therapy: Integrated Care Approach

A care team information sheet will be provided to all staff involved in the care of patients participating in the study—this includes the clinical care team, health care assistants, sports and exercise therapists, dietitians, and occupational therapy technical instructors. This emphasizes the importance of the multidisciplinary approach in supporting patient participants over the 2-year study period and demonstrates the alignment with standard care practice. Such a coordinated effort aims to support patients effectively and ensures the success of the study through consistent and comprehensive personalized care.

### Measurements

The primary outcome is change in body weight, and the physical secondary outcomes include changes in waist circumference, BMI, blood pressure, resting heart rate, and blood cholesterol. All physical health outcomes are routinely collected and will be extracted by the hospital’s business intelligence team at the end of participation. All variables will be compared across a maximum period of 36 months for each participant as follows: up to 12 months prior to enrollment in the study, at baseline, and up to at least 24 months (104 weeks) of treatment.

Each patient’s physical health outcome measures will be evaluated longitudinally, both before and after treatment, to assess the treatment’s impact. Subgroup analyses will also be carried out to determine whether the nature of a patient’s pretreatment physical health history impacted their likelihood of responding to semaglutide therapy. The delayed start nature of the trial will also allow for a comparison of the subgroup of patients who engaged in the nonpharmacological intervention (healthy eating and exercise) with those who were also administered the drug immediately upon enrollment to determine whether exercise and diet can have an impact on patients alone or whether they can only serve as an adjunct to the actions of semaglutide. The patients who are placed on the waiting list for the medication will be monitored for changes in physical health outcomes, as well as additional outcomes such as PA and exercise, appetite and diet, and quality of life. This will essentially have the format of a delayed start study.

All patients will also be monitored for physical health problems as per usual care, particularly T2D, for which semaglutide is prescribed. Blood samples will be taken by a member of the physical health team and sent for analysis every 6 months, and the levels of hemoglobin A_1C_ will be used as indicators of T2D risk. Any patient incidents that staff believe are related to the patients’ participation in the trial will be referenced as such in the Datix risk management information system. This will then be consulted at the end of the study. Recording and reporting of adverse events, including incidents and reactions, will be conducted in accordance with the hospital’s standard clinical practices. The clinical care team information sheet advises staff to reference the patients’ participation in the STEP@STAH study in the Datix record when reporting any incidents they believe may be related to the study. The Datix record will then be consulted at the end of the study. Adverse events and reactions will also be reviewed by the principal investigator and the study steering group (SSG).

In addition to physical health measures, other secondary outcomes will be assessed to determine whether the level of PA and exercise, appetite, and quality of life are affected by the STEP@STAH intervention. For that reason, the following additional tools will be used to collect data that will be correlated with the physical outcomes.

### Control of Eating Questionnaire

Changes in how the individual perceives self-control over their eating behavior (eg, across restraint and discipline, emotional eating, cognitive restraint, and uncontrolled eating) will be explored. While the Control of Eating Questionnaire, which includes 21 items to assess food cravings, is a valid measurement of the experience of cravings [43], the computer-based Leeds Food Preference Questionnaire will also be used to assess food preference to help contextualize the Control of Eating Questionnaire data and explore “liking and wanting component of food preference and food reward” in more detail [44].

### Impact of Weight on Quality of Life–Lite–Clinical Trials Version Health Survey

Changes to health-related quality of life will be assessed using the Impact of Weight on Quality of Life–Lite–Clinical Trials Version. This questionnaire was specifically designed for clinical trials exploring the impact of weight and obesity on quality of life [45]. These will be correlated with the physical health outcomes to determine whether any physical benefit is associated with positive changes in quality of life, particularly in areas most affected by weight-related issues.

### Food, Activity, and Sleep Diary Sheet

The food, activity, and sleep diary sheet will provide a descriptive overview of participants’ lifestyle over the trial period and will be used to provide an indication of how well they adhered to the nonpharmacological aspect of the trial. Diary sheets will be used not only to capture individual experiences [46] but also in conjunction with the activity monitors and PA dashboard to provide an indication of how well the patient engaged with the program. This can then be correlated with the primary physical health outcome of BMI. Although this will be descriptive, it will provide an indication of the adherence of the patient to a quality lifestyle.

### Activity Monitoring Tool and Wearable Tracking Devices

Patients will wear these for 7 days (24 hours per day) every 3 months to monitor their ongoing PA. The data will be downloaded and stored on the participant master log. This will then be correlated with changes in physical health to determine, for example, whether weight loss is associated with an overall increase in PA and exercise. These devices will also monitor sleep patterns and will correlate with patient self-reported sleep in the food, activity, and sleep diary sheet. While these devices will be used to monitor PA, they will also be used as an additional tool to improve BMI and PA as their effectiveness in promoting a healthy lifestyle has been demonstrated in previous lifestyle interventions [47].

### Interviews

Barriers, facilitators, acceptability of the intervention, and potential benefits of the nonpharmacological element will be also explored qualitatively via semistructured interviews from both the patient and staff perspectives. Interviews will be conducted and analyzed by KB, a PhD student.

All additional secondary outcome measures are outlined in a Gantt chart (Table 1) together with a timeline of the interviews. In addition to these, PA data are also routinely collected within the hospital and will be extracted by the business intelligence team and aggregated into weekly figures retrospectively.

**Table 1.** Schedule of additional secondary outcome measures and interviews.

Outcome measures and interviews	Year 1 (month)													Year 2											
	0	1	2	3	4	5	6	7	8	9	10	11	12	1	2	3	4	5	6	7	8	9	10	11	12
COEQ^a^	✓		✓		✓			✓							✓					✓					✓
LFPQ^b^	✓						✓						✓						✓						✓
IWQOL-Lite-CT^c^	✓			✓			✓			✓			✓			✓			✓			✓			✓
Actigraph leap				✓			✓			✓			✓			✓			✓			✓			✓
FAS^d^ diary sheet	✓	✓	✓	✓	✓	✓	✓	✓	✓	✓	✓	✓	✓	✓	✓	✓	✓	✓	✓	✓	✓	✓	✓	✓	✓
Patient interviews	✓							✓					✓												✓
Staff interviews													✓												✓

a COEQ: Control of Eating Questionnaire.

b LFPQ: Leeds Food Preference Questionnaire.

c IWQOL-Lite-CT: Impact of Weight on Quality of Life–Lite–Clinical Trials Version.

d FAS: food, activity, and sleep.

### Statistical Analysis

All outcomes will be analyzed to evaluate the effectiveness and feasibility of the study. As the primary objective is to investigate changes in physical health metrics from baseline until the final follow-up, repeated-measure ANOVA will be used to carry out a multivariate analysis to determine the potential interrelationship between the different variables. Differences in weight change will be assessed using percentages. Changes in various metabolic syndrome components will be assessed using paired 2-tailed *t* tests and Wilcoxon signed-rank tests.

Subgroup analyses will also be carried out to determine whether the nature of a patient’s pretreatment physical health history (diabetes status), their demographic data (biological sex, age, and ethnicity), and their clinical data (primary and secondary diagnosis) impacted their likelihood of responding to semaglutide therapy.

Correlation analysis will be used to identify patterns within the data and specifically to explore the relationship between the level of PA and exercise, appetite, and quality of life and physical health outcomes.

Thematic framework analysis, which is often used in applied research [48], will be used to analyze qualitative data. Through this method, data will be organized and interpreted systematically; therefore, meaningful insights will be identified. Initial codes will be generated from the transcripts to capture meaningful units of data related to barriers, facilitators, and acceptability. Codes will be organized into broader themes and subthemes through iterative review and comparison. Themes will represent patterns and recurring topics across the data. Themes will be interpreted in relation to the research questions, aiming to provide insights into factors influencing the intervention’s implementation and acceptance among patients and staff. The analysis will be conducted by a PhD student, KB, but will be reviewed and discussed with the supervisory team.

### Oversight of the Study

The SSG will meet on a regular basis to oversee the progress of the study and act as study monitor. Responsibilities include reviewing adverse events and protocol deviations and considering the implications and identifying changes that may be required.

The SSG is responsible for providing updates to the hospital medical advisory committee and the medicine management group.

### Ethical Considerations

All data collected as part of this study will be stored securely in a password-protected SharePoint 365 folder accessible only to authorized members of the research team. No personal identifiers will be collected or stored alongside the research data; instead, each participant will be assigned a unique study ID to ensure confidentiality. Interviews will be fully anonymized during transcription. Transcription will be undertaken by the researcher, and audio recordings will be permanently deleted immediately after the transcript has been completed and verified for accuracy. Participant privacy will be protected throughout the research process, and no information that could directly or indirectly identify participants will be included in any publications or reports arising from the study. No compensation or financial incentives will be provided for participation in this study. This project has been approved by the National Health Service Research Ethics Committee (Integrated Research Application System 336454), St Andrew’s Healthcare Service Evaluation and Research Approval Committee (2024/254), and the Loughborough University Ethics Committee (2024-19540-21442). It was registered on December 20, 2024, as a clinical trial on ClinicalTrials.gov (NCT06754163). Written consent will be obtained from participants by a team member before enrolling in the study.

## Results

As of April 2025, a total of 15 participants have been enrolled of the target sample size of 20. The results of the study, which are projected to be obtained by mid-2027, will be shared internally within the hospital where the study is being conducted and will also form part of a PhD thesis. Key stakeholders will receive a short summary of the findings, and all participants are given the option to request the findings of the study in the consent form. Additionally, this study will be submitted to a peer-reviewed academic journal and will also be presented at relevant academic conferences and shared via social media where appropriate.

## Discussion

People with complex mental health conditions being treated in hospitals include patients who are prescribed antipsychotic medications, which can lead to obesity. Many of these individuals have developed or are at risk of developing T2D, a condition that can reduce the life span. The aim of this study is to explore the effectiveness of semaglutide, one of the GLP-1 RAs, in combination with a lifestyle intervention in promoting weight loss and improving metabolic outcomes among individuals with obesity or overweight and SMI who are residing in a psychiatric setting. Specifically, this study seeks to explore whether clinically meaningful reduction in body weight and improvement in other physical health outcomes, such as waist circumference, BMI, blood pressure, resting heart rate, and blood cholesterol, can be achieved for inpatients treated with clozapine or olanzapine. It is anticipated that treatment with semaglutide in combination with a lifestyle intervention will be associated with weight loss and improve other physical outcomes.

It is expected that the findings of this study will be consistent with previous findings in the literature. Clinical studies have shown that semaglutide reduces energy intake; increases feelings of satiety, fullness, and control of eating; reduces feelings of hunger and frequency and intensity of cravings [49]; and reduces the preference for high-fat foods [35]. In addition, the drug has been shown to reduce fasting blood glucose by stimulating insulin secretion and lowering glucagon secretion when blood glucose is high [41,50], which is crucial for preventing complications associated with hyperglycemia [51]. The efficacy and safety of semaglutide for weight management in combination with a behavioral intervention focused on reducing calorie intake and increasing PA has already been evaluated in various STEP trials [32]. Compared with placebo, treatment with semaglutide demonstrated to be effective and clinically meaningful for those who had at least one weight-related comorbidity without T2D [33]. A weight-related comorbidity refers to another medical condition that developed because of excess adiposity in individuals with obesity, such as T2D, obstructive sleep apnea, increased vascular mortality, and cancer [52]. In the STEP trials that excluded participants with T2D, eligible participants had conditions such as dyslipidemia, obstructive sleep apnea, hypertension, or cardiovascular disease [13]. In addition to reduced weight, treatment with semaglutide also significantly improved waist circumference, cardiovascular functioning, and physical functioning compared to placebo [34]. The efficacy of semaglutide was demonstrated regardless of race and ethnicity, age, sex, baseline body weight, BMI, presence of T2D, and level of renal function [53]. All patients were on a reduced-calorie diet and an increased-PA program throughout the STEP trials in addition to having access to individual psychological counseling to help them adhere to the regimen.

While the results of the STEP trials demonstrated the effectiveness of treatment, this was limited to the general population. Given the limited evidence of its effectiveness also in individuals with SMI, there is a need to extend such evidence to a population within a psychiatric setting due to the life span inequity they experience.

It is anticipated that the findings of this study will inform clinical guidelines for health care professionals in managing obesity in people treated with antipsychotics in a secure care setting, ultimately improving their quality of life. Therefore, it is expected that semaglutide will prove its effectiveness as an adjunctive therapy to address obesity in such settings. Additionally, this study aims to contribute to the development of a multicomponent lifestyle intervention for weight management in a secure mental health setting, enhancing the effectiveness of therapeutic treatments while minimizing the side effects of antipsychotic drugs.

While the use of a mixed methods design combining robust quantitative measures with qualitative insights and the length of the study duration are the real strengths of this study as they allow for a comprehensive evaluation, several limitations must also be acknowledged. The study sample is relatively small and was recruited from only 1 mental health hospital, which may limit generalizability. However, this study aims to assess the feasibility and efficacy of the treatment rather than provide conclusive claims. The use of a single-arm design is another limitation of the study as it is difficult to assess whether the results are due to the treatment with medication or the lifestyle intervention. However, this will be further explored qualitatively.

## Abbreviations

AIWG: antipsychotic-induced weight gain
PA: physical activity
RA: glucagon-like peptide-1 receptor agonist
RC: responsible clinician
SMI: severe mental illness
SSG: study steering group
STEP: Semaglutide Treatment Effect in People With Obesity
STEP@STAH: Semaglutide Treatment Effect in People With Obesity intervention protocol modified for a secure mental health setting
T2D: type 2 diabetes
